# Supplementary material for: Multiple bumps can enhance robustness to noise in continuous attractor networks
Source: PLoS Comput Biol. 2022 Oct 10;18(10):e1010547. doi: 10.1371/journal.pcbi.1010547 (PMC9584540; doi:10.1371/journal.pcbi.1010547)
Supplement: S1 Text — (PDF) [file pcbi.1010547.s001.pdf]

S1 Text for "Multiple bumps can enhance robustness to noise in continuous attractor networks"

Raymond Wang[1,2] and Louis Kang[*2]

[1]Redwood Center for Theoretical Neuroscience, University of California, Berkeley
[2]Neural Circuits and Computations Unit, RIKEN Center for Brain Science

Different model parameters

In this section, we revisit many major results for input, spiking, and connectivity noise, but for either a different activation function ϕ (Fig A) or for connectivity strengths W that do not scale with bump number and network size (Fig B). To calculate theoretical predictions for each set of results, we need to substitute the baseline synaptic inputs g into the appropriate equations. They are obtained by running simulations without noise and drive. Notably, the theory still demonstrates close agreement with simulation results under these new conditions.

In Fig A, we use a logistic sigmoid activation function ϕ to convert synaptic inputs g to firing rates s:

$$\phi[g] = \frac{1}{1 + \mathrm{e}^{-g}}. \tag{A}$$

All results with this ϕ are qualitatively identical to those obtained with a ReLU ϕ in the Results section of the main text. To calculate theoretical values, we can no longer use equations from the Results section, which are simplified for a ReLU ϕ. To compute D_{input}, D_{spike}, v_{drive}, and $v_{\text{conn}}(\theta)$, we use Eqs 97, 104, 86, and 108 from the Theoretical methods section of the main text instead.

In the Results section of the main text, we assumed that the connectivity strengths W obey Eq 7 to maintain the same scaled bump shape across bump numbers M and network sizes N. In addition to the theoretical advantages of obtaining simple scaling relationships, this choice can be loosely biologically motivated. Consider the tuning curves of grid cells, which are thought to function as CANs. Their scaled shapes are roughly similar across modules [1], which may differ in bump number [2–4], and across mammalian taxa from rodents to primates [5, 6], whose brains certainly differ in neuron number. This crude observation supports the choice to maintain a fixed scaled bump shape across M and N. Nonetheless, in Fig B, we do not assume Eq 7 of the main text and bump shape invariance. Instead, we fix $w = 0.04$ in Eq 38 of the main text, which fixes the maximum synaptic strength across all networks. This change produces qualitative differences in noise robustness only for circular mapping. Here, under circular mapping, networks with fewer bumps are more robust to all three forms of noise, and larger networks are more robust to connectivity noise. For the corresponding simulations in the Results section of the main text, no major changes in robustness were observed.

*louis.kang@riken.jp

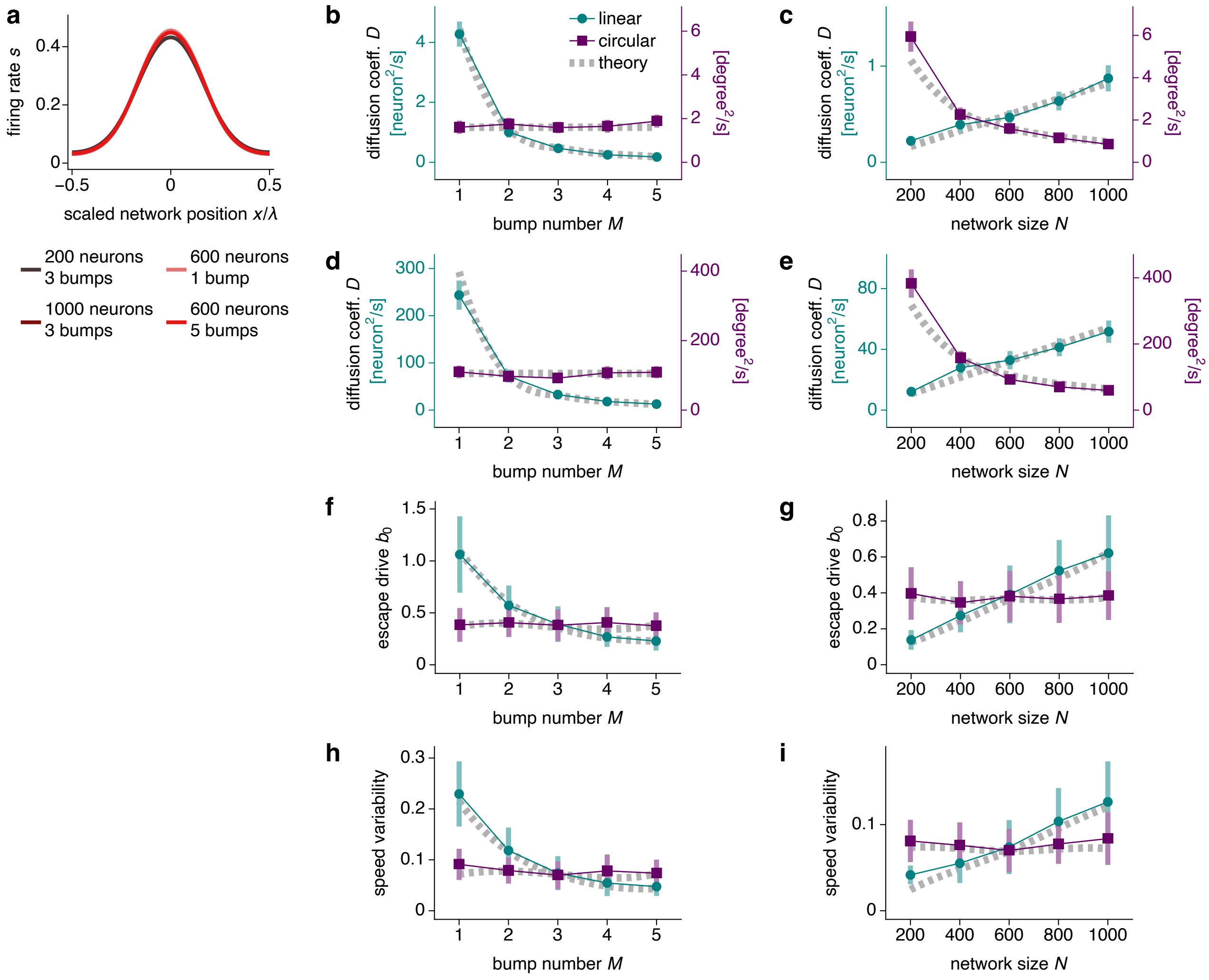

Fig A: Main results repeated for networks with a logistic activation function. (**a**) The scaled bump shape remains invariant across network sizes and bump numbers, accomplished by rescaling connectivity strengths according to Eq 7 of the main text. Curves for different parameters lie over one another. (**b** and **c**) Networks with synaptic input noise. Bump diffusion follows the same qualitative behavior as in Fig 5A and 5B of the main text. (**d** and **e**) Networks with Poisson spiking noise. Bump diffusion follows the same qualitative behavior as in Fig 5C and 5D of the main text. (**f**–**i**) Networks with connectivity noise. (**f** and **g**) Escape drive follows the same qualitative behavior as in Fig 7D and 7E of the main text. (**h** and **i**) Bump speed variability follows the same qualitative behavior as in Fig 8D and 8E of the main text. The activation function ϕ takes the form in Eq A. In **f**–**i**, we use connectivity noise of magnitude 0.003. In **h** and **i**, we use drive $b = 2.5$. The rest of the parameters are identical in value to those used in the main text.

Additional information results

Fig C shows additional mutual information analyses in networks with input noise. We choose two new linear coordinate ranges, and we use large networks of size $N = 2000$ to explore bump numbers up to $M = 32$. In Fig Ca, we use 2000 cm as the range, which is the maximum value according to the linear mapping in Fig 4A of the main text. Bump ambiguities exist for all networks with bump number $M > 1$. Nevertheless, mean single-neuron mutual information remains largely constant across M. Thus, the effects of decreased bump diffusion and increased bump ambiguity at larger M counteract each other.

In Fig Cb, we compare each network's encoding of a linear coordinate range that is equivalent to its

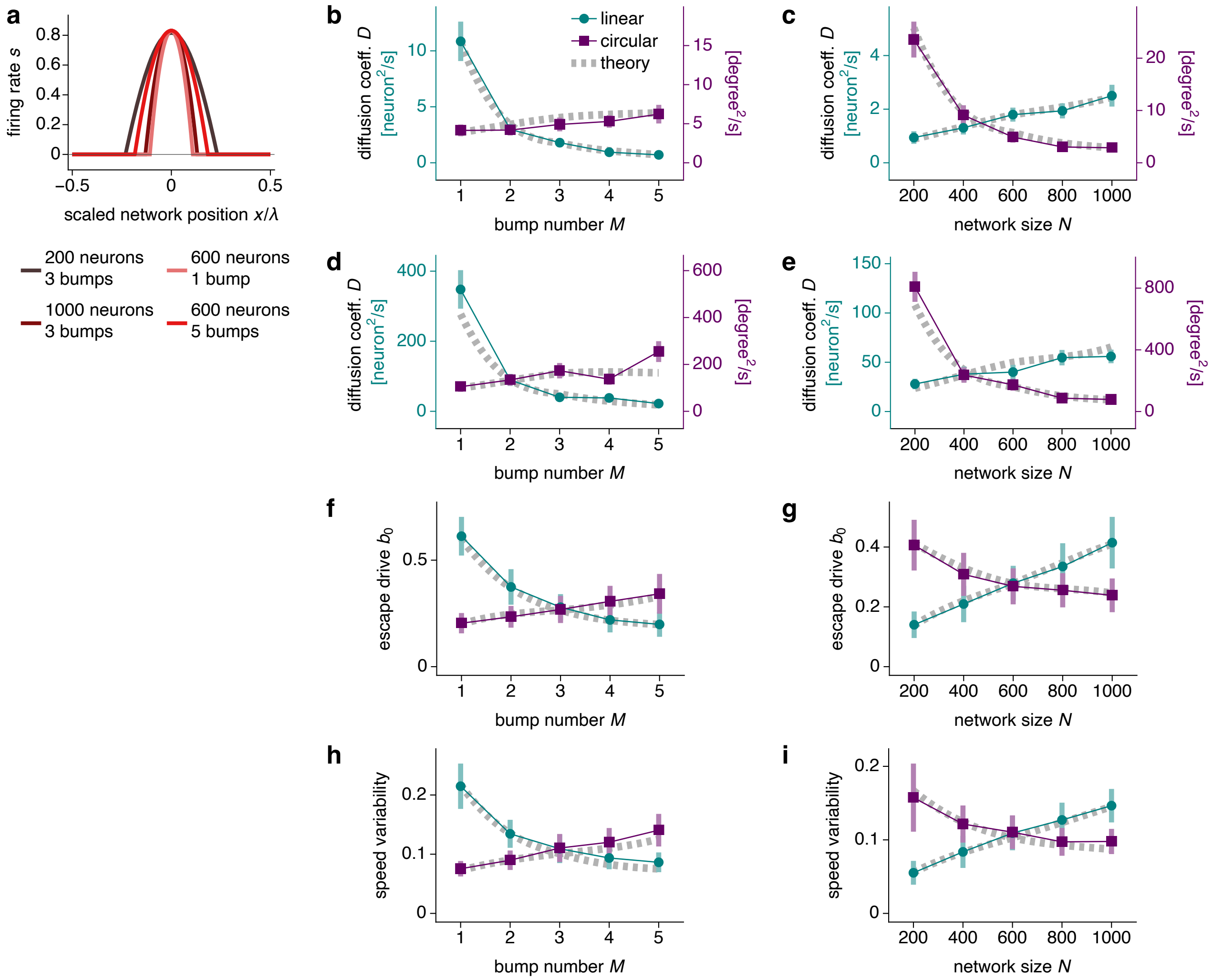

Fig B: Main results repeated for networks without rescaling of connectivity strengths according to Eq 7 of the main text. (**a**) The scaled bump shape no longer remains invariant across network sizes and bump numbers. (**b** and **c**) Networks with synaptic input noise. Bump diffusion follows the same qualitative behavior as in Fig 5A and 5B of the main text, except that here it slightly increases with bump number under circular mapping. (**d** and **e**) Networks with Poisson spiking noise. Bump diffusion follows the same qualitative behavior as in Fig 5C and 5D of the main text, except that here it slightly increases with bump number under circular mapping. (**f**–**i**) Networks with connectivity noise. (**f** and **g**) Escape drive follows the same qualitative behavior as in Fig 7D and 7E of the main text under linear mapping. It slightly increases with bump number and decreases with network size under circular mapping. (**h** and **i**) Bump speed variability follows the same qualitative behavior as in Fig 8D and 8E of the main text under linear mapping. It slightly increases with bump number and decreases with network size under circular mapping. The connectivity W still takes the form in Eq 38 of the main text, except that here we fix $w = 0.04$ across all bump numbers and network sizes. In **h** and **i**, we use drive $b = 1.0$. The rest of the parameters are identical in value to those used in the main text.

bump distance $\lambda = N/M$. Therefore, there is never bump ambiguity, but mutual information also remains relatively constant across M. As described in Simulation methods of the main text, mutual information is computed using 20 coordinate values evenly spaced across each range. At larger M, the spacing between coordinate values decreases along with the coordinate range. Distinguishing among positions at finer scales is a more difficult task and cancels the effect of decreased bump diffusion. Thus, noisy networks with more or fewer bumps are equally capable of encoding linear coordinates at proportionally finer or coarser scales. In fact, this scaled linear coordinate range is identical to a circular coordinate range of 360°, so these linear

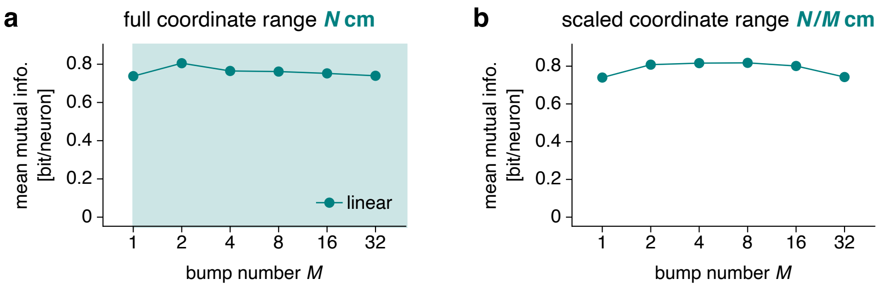

Fig C: Mutual information between neural activity and linear coordinates for additional coordinate ranges and bump numbers M. Networks contain input noise of magnitude $\sigma = 0.5$. (**a**) Full coordinate range of $2000\,\mathrm{cm}$ in networks of size $N = 2000$. Mutual information remains largely constant across bump number. In the shaded region, the coordinate range exceeds the bump distance. (**b**) Scaled coordinate range that remains equivalent to the bump distance N/M in networks of size $N = 2000$. Mutual information remains largely constant across bump number. The rest of the parameters are identical in value to those used in the main text. Points indicate data from 96 replicate simulations at each coordinate value averaged over neurons and bars indicate bootstrapped standard errors of the mean.

results are equivalent to the circular results in Fig 6D of the main text.

Splitting networks

Suppose we have a network of size N with M bumps. Consider splitting it into n separate networks, each of size $N' = N/n$ with $M' = M/n$ bumps. How would the combined readout of these split networks compare to the intact network in terms of noise robustness? We will address this question for input and spiking noise, assuming invariance of the scaled synaptic inputs $g(x/\lambda)$ over M and N (Fig 2F of the main text), which then permits the scalings for diffusion coefficients and velocities in the main text to hold.

Input noise and spiking noise produce diffusion. Using Eqs 15 and 21 of the main text, the diffusion coefficient for each split network D' compared to that of the intact network D is given by

$$D' = nD \tag{B}$$

for both input and spiking noise and both linear and circular mapping. Now let's consider a simple combined readout of bump position for the split networks that simply averages over the bump position of each network $\mu = 1, \ldots, n$:

$$\bar{\theta}(t) = \frac{1}{n} \sum_{\mu} \theta^{\mu}(t). \tag{C}$$

For any network, the diffusion coefficient D describes the variance in bump position θ across replicate simulations. According to Eq 91 of the main text,

$$\mathrm{Var}[\theta(t)] = 2Dt, \qquad \mathrm{Var}[\theta^{\mu}(t)] = 2D't, \qquad \mathrm{Var}\big[\bar{\theta}(t)\big] = 2\bar{D}t, \tag{D}$$

where D, D', and $\bar{D}$ are diffusion coefficients for the intact network, each split network, and the combined readout, respectively. Then,

$$2\bar{D}t = \mathrm{Var}\big[\bar{\theta}(t)\big] = \frac{\mathrm{Var}[\theta^{\mu}(t)]}{n} = \frac{2D't}{n} = 2Dt. \tag{E}$$

Thus, the diffusion coefficient for the combined readout of the split networks is equal to that of the intact network.

In the case of connectivity noise, a simple comparison between split and intact networks cannot be made. The split networks contain fewer noisy synapses in total: $4N^2/n$ compared to the $4N^2$ entries of the noisy connectivity matrix $V_{\alpha\beta}(x, y)$ for the intact network. One can analyze the average readout of bump velocity across the split networks

$$\bar{v}(t) = \frac{1}{n} \sum_{\mu} v^{\mu}(t), \tag{F}$$

but with the discrepancy in the amount of noise and additional assumptions required to connect $v(t)$ with $v(\theta)$, which is the predominant form of v in our formulas, we will not continue further.

References

1. Stensola H, Stensola T, Solstad T, Frøland K, Moser MB, Moser EI. The entorhinal grid map is discretized. Nature. 2012;492(7427):72–78. doi:10.1038/nature11649.

2. Gu Y, Lewallen S, Kinkhabwala AA, Domnisoru C, Yoon K, Gauthier JL, et al. A map-like micro-organization of grid cells in the medial entorhinal cortex. Cell. 2018;175(3):736–750. doi:10.1016/j.cell.2018.08.066.

3. Kang L, Balasubramanian V. A geometric attractor mechanism for self-organization of entorhinal grid modules. eLife. 2019;8:e46687. doi:10.7554/elife.46687.

4. Khona M, Chandra S, Fiete IR. From smooth cortical gradients to discrete modules: spontaneous and topologically robust emergence of modularity in grid cells. bioRxiv. 2022; p. 2021.10.28.466284. doi:10.1101/2021.10.28.466284.

5. Killian NJ, Jutras MJ, Buffalo EA. A map of visual space in the primate entorhinal cortex. Nature. 2012;425(7426):184. doi:10.1126/science.1069590.

6. Jacobs J, Weidemann CT, Miller JF, Solway A, Burke JF, Wei XX, et al. Direct recordings of grid-like neuronal activity in human spatial navigation. Nat Neurosci. 2013;16(9):1188–1190. doi:10.1038/nn.3466.
