# Supplementary material for: Multiple bumps can enhance robustness to noise in continuous attractor networks
Source: PLoS Comput Biol. 2022 Oct 10;18(10):e1010547. doi: 10.1371/journal.pcbi.1010547 (PMC9584540; doi:10.1371/journal.pcbi.1010547)
Supplement: Attachment — Submitted filename: Response.pdf [file pcbi.1010547.s002.pdf]

RIKEN Center for Brain Science
2-1 Hirosawa, Wako-shi, Saitama, 351-0198 Japan
Tel: +81-48-462-1111
https://cbs.riken.jp/en

June 27, 2022

Dear *PLOS Computational Biology* Editors and Reviewers:

My colleague Raymond Wang and I are pleased to submit a revision of our manuscript, "Multiple bumps can enhance robustness to noise in continuous attractor networks." We appreciate the thoughtful comments from the Reviewers, and below, we detail how we have fully addressed all their concerns with new text and three new figures. In doing so, our manuscript has become stronger with more comprehensive results and a more engaging narrative.

Reviewer 1 *(Although Reviewer 1's comments were not described as Major and Minor points, we have taken the liberty to do so for ease of referencing.)*

Major point 1: "My primary issue with the work is that the coupling strength scaling (Eq. 13) the authors choose seems to be doing a lot of the work in shaping these relations, but this is not well justified."

We thank the Reviewer for the opportunity to clarify our circular mapping, of which the coupling strength scaling is a central component. As argued in line 190 of text, we find this scaling natural for circular mapping because it establishes consistent path integration behavior in units of degrees. We alternatively present all our results under a trivial linear mapping which does not include coupling strength scaling nor apply the conversion factor of Eq. 12, which is the other central component of our circular mapping. Again, we find it natural for Eqs. 12 and 13 to be included or excluded together to maintain consistent path integration in one physical unit or another.

But indeed, it's possible to ask how our results would change without coupling strength scaling (Eq. 13), while still applying the conversion factor of Eq. 12. Our results for connectivity noise under circular mapping would become identical to those for linear mapping because coupling strength scaling is the sole factor responsible for the observed differences. However, our results for input and spiking noise would not change because diffusion is independent of coupling strength. To help the reader understand these points, we've added new text at lines 228 and 324.

Major point 2: "Moreover, I do not see how general this result is or if you've simply chosen constituent functions of the model (\phi and W) that are non-generic and so produce a behavior that would not likely persist in other networks with different transfer or weight functions. […] I don't think the authors are really hiding anything, but I would like to see how well these results hold up across a broader range of model function choice. […]"

We agree that reproducing our main results for different choices of ϕ and W would better demonstrate the power of our model. We have done so with a new Appendix section and two new figures, Fig. 9 and Fig. 10. In Fig. 9, we use a logistic sigmoid ϕ, which differs substantially from a ReLU ϕ in that it is a saturating function. In Fig. 10, we follow the Reviewer's suggestion in Minor point 3 to do away with connectivity strength scaling. For both these modifications, our results are mostly qualitatively unchanged under all three forms of noise. The only differences appear under the combination of W scaling removed and circular mapping applied; in that case, robustness to all three forms of noise becomes inversely related to bump

number and robustness to connectivity noise becomes directly related to network size (instead of exhibiting no strong dependence). Importantly, our conclusion that robustness to noise increases with bump number for linear coordinates is unchanged under all parameter choices. Furthermore, our theory still demonstrates good quantitative agreement with our simulation results.

Minor point 1: "You seem to suggest that networks with M bumps essentially code information the same way regardless of bump number. However, a single neuron will see activity rise and fall M times in a "rotation period" and so the same velocity integration process can represent an interval of 1/M the length. This is made fairly clear in past papers of grid cell models that the authors cite. It is unclear how bump identity could be known by a single neuron (in Fig. 1). It seems like you would shorten the period. What is driving the rotation of the bumps? Is it now that you have to pack neurons of a particular tuning N times as tightly?"

Indeed, there is no single obvious way through which a network's information encoding varies (or not) with bump number M. Therefore, we presented all of our results under two different ways, which are linear and circular mapping. Indeed, under linear mapping, which may apply to grid cells, the period would be shortened by increasing M because bump identity is not known by individual neurons. We have clarified this point by adding new text at line 178. Moreover, our new mutual information analysis in Fig. 5 explicitly demonstrates how coarse local cues can help networks resolve the ambiguity among multiple bumps. As for packing neurons more tightly, we believe that the circular mapping captures that idea because each neuron maintains the same (unimodal) tuning across all bump numbers and network sizes. The driving input b, coupled to the network with strength γ, drives the rotation of the bumps.

Minor point 2: "The intro is wrong in asserting no one else has examined the effect of bump number of noise-induced WM degradation. Many past papers have: […]"

We are grateful to the Reviewer for mentioning these references! We have attempted to find all previous related work through a thorough literature search, but we neglected to look for papers related to working memory. We now cite these papers in lines 52 and 384 and compare their results with ours. Interestingly, they generally find a trend opposite to that demonstrated in our work, and we discuss how networks designed for multiple-item working memory and those designed for path integration can differ. We also clarify in line 62 that the relationship between bump number and noise robustness has only been previously unexplored for path-integrating networks.

Minor point 3: "Per my point above, what justifies rescaling connectivity as in Eq 7? A lot of results in the paper hinge on this, but it is not well justified."

We have addressed this important point in our response to Major point 2.

Minor point 4: "Provide justification/citation for the reduction from Eq 1 to Eq 3. Does this depend on the ReLu transfer function assumption? You do this in the Theoretical model section too, but don't really prove the reduction is valid."

This reduction is achieved through Eqs. 36 and 37. For small connectivity shift ξ, the difference in W between the two populations is small, so the noiseless and driveless dynamical equation does not depend

on the population identity to linear order in ξ. The reduction does not depend on the form of the activation function, and now make this point clear with new text in line 428.

Minor point 5: "Line 119: Doesn't the period of the network impact the spacing more than a small difference? Seems like this would be the case especially if L/\lambda equals an integer plus a half."

Indeed, we did not address that case, which can impact the spacing by ~50% for a network with 1-2 bumps. Thus, we have removed the word "small" and rephrased the sentence in terms of integral bump numbers (now line 124).

Minor point 6: "Why does keeping the bump shape the same make for a fair comparison? Neural architecture would seem more likely to be the constrained quantity. You should provide more evidence that there is some straightforward mechanism for the amplitude of neural connectivity to be varied so that bump are of the same size regardless of their number. This seems like it would be a complicated feedback control mechanism."

We have addressed this important point in our response to Major point 2 and Minor point 3. In Fig. 10, we have repeated all our results for a connectivity matrix in which the amplitude is fixed. In the corresponding text in the Appendix, we provide additional loose motivation for keeping the bump shape invariant, arising from the roughly similarly shaped tuning curves of grid cells across scales and animals. This does not attempt to address the question of mechanism that the Reviewer asks. Yet, Fig. 10 does demonstrate the robustness of our results, which are mostly qualitatively unchanged except in relation to network size under circular mapping.

Minor point 7: "You introduce the notion of linear vs circular coordinates, but you are not very clear about what boundary conditions you use in the linear coordinate case. Are they free boundaries? In general you are on clear about what weight function W is used in any of the plots. Is this somewhere buried in the methods at the end?"

We only consider linear vs. circular coordinates in terms of mapping from network coordinates. The same periodic ring networks illustrated in Fig. 2A are mapped to both of these types of physical coordinates. We now emphasize this point in line 201. Furthermore, we follow the Reviewer's good suggestion to describe our use of general vs. specific W in line 97, as well as reiterating this point in line 438.

Minor point 8: "Line 355: You say most of your results apply to a general nonlinear transfer function. Where do you make it clear that this is true? All your plots are for the ReLU, so I'm not sure where you're showing results for other nonlinearities. Do Eq. 8, 10, 12 all hold for general \phi or specifically the ReLU? Make this more clear."

We also follow this helpful suggestion and clarify our use of general vs. specific ϕ in line 90, as well as in line 428.

Minor point 9: "Your analysis assumes periodic solutions g that are sinusoidal, but shouldn't there also be solutions that might just be single localized bumps? Did you explore this possibility? Is it clear that choosing W as in Eq. 37 rules out such solutions? If so, it seems to me that the W you chose is nongeneric, since typically in continuum bump attractor networks we should find single bumps with quiescence around."

If the period λ equals the network size N, the solution corresponds to a single-bump configuration. We have clarified this point in line 421.

Reviewer 2

Major point 1: "Although I largely agree with the author's finding that drift due to fast noise can be reduced by having more bumps, I do not find that this necessarily confers, or that the authors show that it confers, a benefit when looked at from the perspective of the presumed function of continuous attractors. Having more bumps in a network of N neurons, in addition to reducing noise, reduces the resolution by which the external covariate can be encoded. In other words, what the authors do not address is how much "information" is lost or maintained as a result of drift with different number of bumps. […] I think the authors should perhaps use mutual information to study this tradeoff as has been used e.g. in Roudi and Treves PLoS Comp Biol 2008."

We take the Reviewer's good suggestion that we consider mutual information an alternative metric for network performance. In our new Fig. 6, we calculate the mutual information between a physical coordinate encoded by the network, either linear or circular, and single-neuron activities; we then average over all neurons in the network. Ideally, we would want to calculate the mutual information between the coordinate and the joint activities of all the neurons, which confer much more information than single neurons, but the curse of dimensionality makes it computationally intractable to estimate such probability distributions. An alternative way of reducing dimensionality is to project the network activity along attractor states, as performed in Roudi & Treves 2008, but we choose to simply consider single-neuron activities.

For narrow coordinate ranges, our mean mutual information analysis precisely corroborates our diffusion constant analysis, with an inverse relationship between the two metrics across all mappings, bump numbers, and system sizes. In the case of linear coordinates, we can ask the network to encode coordinate ranges that are wider than that which can be uniquely encoded by the network. In this case, ambiguities arise among the different bumps and the mutual information declines. However, coarse local cues can be combined with neural activity to resolve the ambiguities and rescue the advantage of multiple attractor bumps towards boosting mutual information. In our original manuscript, we commented that such cues can be used to resolve ambiguities; here, thanks to the Reviewer's suggestion to consider mutual information, we can explicitly see how they do so.

Major point 2: "The only focus on the effect of fast noise on the drift. If not more important, an equally important problem, is that of the noise induced by quenched noise, e.g. when the connectivity is not a perfect connectivity in Fig2 b but with a temporally fixed noise added to it, e.g. see Eq. 2 Itskov et al 2011. I think the authors should discuss the interaction between multi- bumps solutions and the influence of quenched noise in more detail perhaps also discussing the recent result in https://arxiv.org/pdf/2109.03879.pdf"

We have in fact devoted much of the paper to topic of quenched connectivity noise. Figs. 7 and 8 (formerly 6 and 7), the subsection "More bumps improve robustness to connectivity noise under linear mapping" in the Results section, and the subsection "Drift velocity $v_{\mathrm{conn}}(\theta)$ due to quenched connectivity noise" in the Theoretical methods section explore this topic. We have also cited the Itskov, et al. paper in multiple places. The Can & Krishnamurthy paper is interesting and we now cite it on line 384, but unfortunately our framework does not readily apply to their work. Their recurrent neural networks crucially involve general, unstructured connectivity and multiplicative, gating-type coupling between neurons, both of which are not captured in our theoretical development.

Minor point 1: “There is also a minor issue regarding the way the authors discuss CANs. The authors seem to confuse evidence that is compatible with CANs with evidence that shows the existence of CANs in the brain. As far as I can say, none of the papers cited by the authors show that CANs are actually implemented in the brain or are actually used by the brain. The cited evidence are either theoretical models or experimental data that are consistent or supportive of CANs. I suggest the authors be more careful (e.g. "The brain uses path-integrating CANs" can be changed to "Path integrating CANs have been proposed as a mechanism ...").”

We have made this suggested change in line 36. Our original wording was meant to reflect that, at least in the case of the *Drosophila* central complex, CAN dynamics are very well-established. However, we agree that the evidence in mammalian grid cells, although mounting, is indirect and not conclusive. We now appreciate that our original wording could be interpreted to be stronger than we had intended.

Our new manuscript has addressed all the Reviewers’ comments and is consequently stronger, more refined, and better connected with previous literature. We believe that your readers will enjoy and readily engage with our findings. Also, we have now uploaded our simulation code onto the repository https://louiskang.group/repo; we wanted to prepare this revision before uploading it. Please do not hesitate to contact me if you have any further questions and/or comments.

Sincerely,

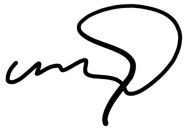

Louis Kang
Unit Leader, Neural Circuits and Computations Unit
RIKEN Center for Brain Science
louis.kang@riken.jp
