# Supplementary material for: Multiple bumps can enhance robustness to noise in continuous attractor networks
Source: PLoS Comput Biol. 2022 Oct 10;18(10):e1010547. doi: 10.1371/journal.pcbi.1010547 (PMC9584540; doi:10.1371/journal.pcbi.1010547)
Supplement: Attachment — Submitted filename: Response.pdf [file pcbi.1010547.s003.pdf]

Center for Brain Science

RIKEN Center for Brain Science
2-1 Hirosawa, Wako-shi, Saitama, 351-0198 Japan
Tel: +81-48-462-1111
https://cbs.riken.jp/en

August 12, 2022

Dear *PLOS Computational Biology* Editors and Reviewers:

My colleague Raymond Wang and I have enclosed a second revision of our manuscript, “Multiple bumps can enhance robustness to noise in continuous attractor networks.” We are pleased that our previous revision satisfied Reviewer 1, who now supports our manuscript for publication. We hope that Reviewer 2 would share a similar opinion, even though a response was not provided. This revision addresses the points raised by the new Reviewer 3. In doing so, we have reorganized and augmented our Appendix into a new Supporting information document. This way, we can comprehensively respond to the Reviewers’ concerns while keeping the main text focused and easily readable for your general computational audience.

Reviewer 1: All points addressed in the last revision

Reviewer 2: No response was provided

Reviewer 3: We are delighted that Reviewer 3 described our paper with the statements “Wang and Kang give a convincing exposé,” “the figures are polished and easy to understand, and the text well-written,” and “the analysis is elaborate and the theory sound.” Below, we respond to the Reviewer’s Points in detail.

Point 1: “In particular, explaining more explicitly how the choice of mapping relates to the results and why multiple bumps and a low number of neurons is beneficial for a linear mapping but not a circular one would make it easier to understand the non-triviality of the findings and thus the impact of the paper. E.g. doubling the number of bumps doubles the number of neurons encoding each (fixed) location under both linear and circular mapping. I.e. going from one to two bumps is similar to splitting the population into two networks, each encoding half the range of the original network but with equal intervals between each encoded position in the linear case, and the full range for circular mapping, but with twice the interval between each angle. Could the results thus depend on the number of neurons encoding each (fixed) interval, the size of the interval, the total range of coordinates encoded by the network or perhaps just the "spread" of noise across bumps?”

Indeed, a more descriptive, intuitive understanding of mapping and noise robustness would bolster our results. We heed the Reviewer’s encouragement and explain in line 242 how the scaling for diffusion coefficients may be intuitively conceptualized. Our argument implicitly alludes to concepts that the Reviewer has mentioned, such as coordinate ranges, but instead of sizes of intervals, it invokes single neuron tuning curves and Fisher information, which are also relevant to the Reviewer’s Point 4. Fisher information has been shown to be related to mutual information as well as to bump diffusion in CANs. As described in Burak & Fiete (2012), internal Fisher information of a CAN depends only on the number of neurons in the network and their tuning curves. For circular coordinates, tuning curves are constant across bump numbers M and network sizes N, so Fisher information should be simply proportional to N and independent of M. For linear coordinates, the steepness of tuning curves is inversely proportional to the bump distance $\lambda = N/M$, so Fisher information should be simply proportional to M^2/N. Then, invoking the inverse proportionality between Fisher information and

diffusion coefficients discovered by Burak & Fiete (2012), we obtain our scaling results. We hope this argument captures the spirit of the Reviewer's Point in that noise-related scalings and coordinate mappings can be connected by considering simple features of the network. Furthermore, in line 202, we mention how intermediate mappings between the linear and circular cases with power law scalings can be implemented. Perhaps by considering a broader spectrum of coordinate mappings, the reader can develop better intuition for the relationships among them.

Point 2: "How do the results compare when splitting the networks - is the combined readout of multiple networks more or less robust than the readout of one network with multiple bumps and which is more biologically plausible?"

We analyze the Reviewer's nice suggestion in Supporting information. For diffusion produced by input and spiking noise, a simple averaged readout of bump position from split networks is equally robust as the position of the intact network. Unfortunately, connectivity noise cannot be easily compared because splitting networks vastly decreases the total number of noisy synapses.

Point 3: "Lines 130-131 say the scaling of W removes any influence of bump shape. However, the change of firing rate over network position (i.e. the slope of the bump ds/dx ~ M/N = 1/\lambda), seems to be an important factor for robustness (e.g. eq. 10). It would seem the results could be explained by saying that adding more bumps (in the linear case) decreases the bump distance, increasing ds/dx (hence changing the bump shape) and thus increasing robustness (while under circular mapping ds/dx is unchanged as the network distance is scaled)?"

Yes, the Reviewer is correct that the bumps are indeed stretched across different network sizes and bump numbers, which drives the changes in noise robustness. We intended for "influence of bump shape" to refer to non-affine changes other than simple stretching, but we see how that phrasing is confusing. We have thus revised line 130.

Point 4: "Similarly, it could be explicitly shown in Fig. 6 how the temporal/spatial shape of the bumps changes when introducing more bumps and how this leads to a different spatial tuning curve per neuron and thus a higher mutual information score for the linear case. In this regard, I am also missing mutual information scores in Fig. 10."

The Reviewer makes a good suggestion to consider the spatial tuning curves and their connection to information-related quantities. As mentioned in our response to Point 1, we now connect tuning curve shape with Fisher information in line 238, which is related to both mutual information and diffusion coefficients of CANs. As for Fig. 10 (now Fig. S2), we agree with the Reviewer on the importance of mutual information. We find that it is best explained with a comprehensive set of coordinate arrangements like in Fig. 6, because these choices can have significant impact (the Reviewer alludes to this idea in Point 5). We intend Fig. 10 (now Fig. S2) to demonstrate that the network demonstrates similar noise-related dynamics under both logistic sigmoid and ReLU activation functions. To do so, we find it better to use quantities more closely related to the dynamics, such as diffusion coefficients and bump velocities, than mutual information.

Point 5: "In computing mutual information, I am wondering if it would be fairer to look at the whole coordinate range of the network? Looking at different number of bumps is similar to studying neurons of grid cell modules of different scales and I would expect the descriptive power of each neuron to be the same but at different spatial scales. Visual cues (or multiple modules) may be involved in confining the possible states, but would the results then be obvious in that grid modules with small spacing gives a more precise description of location (in a short interval) than those with large spacing?"

We take the Reviewer's suggestion to augment our mutual information results, which depend strongly on both the range and resolution of coordinate values used in our analysis. In Fig. S3A, we use the whole coordinate range and explore bump numbers up to 32. We find that mutual information does not substantially change with bump number, in accordance with Fig. 6D. In Fig. S3B, we employ a scaling such that the coordinate range and interval between coordinate values are proportional to bump distance. We find that mutual information is relatively constant across bump number, which supports the Reviewer's prediction that "the descriptive power of each neuron [would] be the same but at different spatial scales." In fact, this scaling of a linear coordinate is precisely how we treat circular coordinates in Fig. 6D.

<u>Point 6</u>: "It would also be interesting to see how robust the readout of the combined network of grid cell modules (of different spacing) would be with a varying number of bumps and how they could interact to resolve the ambiguity."

Indeed, combining the readout of multiple modules (with or without interactions between them) would be very interesting. We are considering doing so for a future manuscript, and we feel that it is beyond the scope of this one.

We hope our second revision, which carefully addresses new concerns, further enhances our manuscript's suitability for publication. Please do not hesitate to contact me if you have any further questions and/or comments.

Sincerely,

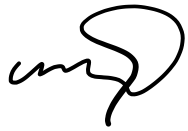

Louis Kang
Unit Leader, Neural Circuits and Computations Unit
RIKEN Center for Brain Science
louis.kang@riken.jp
